# Tuberculous Milk: Report of the Royal Commission

**Published:** 1896-04-25

**Authors:** 


### The Hospital

An Institutional Journal of

The Medical Sciences and Hospital Administration, with

AN EXTRA NURSING SUPPLEMENT.

Vol. XX.—No. 500.] [April 25, 1896.

### Tuberculous Milk: Report of the Royal Commission.

A Royal Commission is an excellent method of eliciting valuable information. But what one always feels about it is that there is no correspondingly excellent method in use for making the information, when elicited, seive the purpose it is so well designed to serve. The Royal Commission on Tuberculosis, whose final report has recently been issued, had for its object "To inquire and report what is the effect, if any, of food derived from tuberculous animals on human health; and, if prejudicial, what are the circumstances and conditions with regard to the tuberculosis in the animal which produce that effect upon man." This is as practical and as widely important an object as could well be desired. The information contained in the present Report is singularly full and complete, and the evidence, set forth in the form of question and answer, is of a kind which the most ordinary intelligence can study with interest and master competently. And yet the progress made in the incorporation of the knowledge into the social economy and general habits of the people will probably be as slow and as difficult as the spread and establishment of a reasonable and highly moral religion. It seems to be a peculiarity of human nature that the higher the knowledge which is offered for its acceptance the slower and the more unwillingly is that knowledge accepted and turned to practical account.

We may pick out for our present purpose, from the pages of the Report, the case of tuberculous milk. Milk is one of the foods "derived from tuberculous animals" which it was a part of the special business of the Commission to inquire about. Now according to the evidence of Dr. German Sims Woodhead, F.R.S.E., Director of the Laboratories of the Conjoint Examining Board in England of the Royal Colleges of Physicians and Surgeons, cows' milk is very liable indeed to become the vehicle of tuberculosis, and young children, whose diet consists, or ought to consist, very largely of cows' milk, are at a period of life when the susceptibility to tuberculosis and other forms of infectious disease is at its maximum. Here, then, we have a conjunction of circumstances in which lies the possibility of the loss of innumerable lives, and of the destruction of the happiness of large numbers of parents. And yet, according to the evidence of the same scientist, tuberculosis is absolutely and easily preventable, not only in healthy children, but even in those who are weakly, and have an inherited tendency to tuberculosis, by the simple method of keeping the children away from the possibility of contagion by the Bacillus tuberculosis. In other words, tuberculosis, in one or other of its forms, is at the present time one of the most common and deadly of children's diseases, whereas, if we did but put into practice the science which we possess, it ought to be almost entirely unknown.

If these things be so, and they are, it is manifest that a people which pretends to any science at all should be able to deal with them effectually, and to rid the country very largely of infant tuberculosis. With us, at any rate as yet, not very much has been done in this direction. In truth, we are are not a very scientific people, preferring what we call "practice" to science. But the science of the really scientific man is practice in these modern days. The members of the Commission kept the witnesses to the practical aspect of things with an insistence which must have been almost exasperating to them. If things be as you affirm, they said in effect to Dr. Sims Woodhead, what advice of a practical nature do you give the Commission in order that tuberculosis may be as far as possible stamped out in our own country? By way of answering that question Dr. Woodhead gave a detailed account of the methods employed, and employed successfully, at Copenhagen and elsewhere in Denmark. It is a little humiliating for us to learn that our progressive and learned Empire is behind so inconsiderable a nation as Denmark in a matter of practical everyday science; but such is the case. Denmark has gone beyond us almost out of sight in this question of the application of the discoveries of science to one of the most distressing sets of facts in common life.

An important question to ask at the present juncture is this: Whether the State should be called upon to secure the application of the discoveries of science to every-day affairs, or whether private enterprise should take the initiative. It will surprise no thoughtful person to learn that in Denmark private enterprise has altogether outrun State action in this instance; and that to private enterprise are due by far the most effectual steps which that country has yet taken towards the stamping-out of tuberculosis among the people. Some ten or twelve years ago a large association, consisting entirely of private persons, was formed in Copenhagen for the purpose of securing to the inhabitants an abundant supply of milk and meat which should be entirely free from the taint of tuberculosis. The association is in direct communication with farmers; and by its rules every farmer has a distinct interest in supplying non-tuberculous milk. A system of inspection by properly qualified veterinary surgeons is carried out, constantly. Every cow which is milked for the association is examined at least once a fortnight. Cows which are in a suspicious condition are removed from the milking herd. But—and this is the chief point in the system—the farmer is paid for the full daily yield of milk until it is definitely ascertained whether a suspected animal be actually tuberculous or not. If in the long run it is proved to be tuberculous, it is killed, and the farmer, so far as the Association is concerned, has to stand the loss. This system is found to work with perfect success, because the Association guarantees a regular demand, with full market prices, so long as nothing but perfectly pure milk is supplied. A regular market of this kind is of such infinite importance to the milk producer that it is found by experience that tuberculous milk is practically never supplied to the Association at all. Our space is done; but the argument may be summed up in a sentence. It is as plain as possible to the scientific mind that private enterprise, or private enterprise supplemented by municipal action, may rid the country almost entirely of tuberculosis from milk whenever the country pleases to be at the necessary trouble and cost.

